# Tetrahedral framework nucleic acid–based small‐molecule inhibitor delivery for ecological prevention of biofilm

**DOI:** 10.1111/cpr.13678

**Published:** 2024-05-29

**Authors:** Yuhao Liu, Kechen Li, Weijie Zhuang, Lulu Liang, Xiangyi Chen, Dongsheng Yu

**Affiliations:** ^1^ Hospital of Stomatology, Guangdong Provincial Clinical Research Center of Oral Diseases, Guangdong Provincial Key Laboratory of Stomatology, Guangdong Key Laboratory for Dental Disease Prevention and Control Sun Yat‐Sen University Guangzhou China; ^2^ Guangzhou Development District Hospital, Chinese Association of Medicinal Biotechnology Southern Center of Biology Diagnosis and Therapy Guangzhou China

## Abstract

Biofilm formation constitutes the primary cause of various chronic infections, such as wound infections, gastrointestinal inflammation and dental caries. While preliminary achievement of biofilm inhibition is possible, the challenge lies in the difficulty of eliminating the bactericidal effects of current drugs that lead to microbiota imbalance. This study, utilizing in vitro and in vivo models of dental caries, aims to efficiently inhibit biofilm formation without inducing bactericidal effects, even against pathogenic bacteria. The tetrahedral framework nucleic acid (tFNA) was employed as a delivery vector for a small‐molecule inhibitor (smI) specifically targeting the activity of glucosyltransferases C (GtfC). It was observed that tFNA loaded smI in a small‐groove binding manner, efficiently transferring it into *Streptococcus mutans*, thereby inhibiting GtfC activity and extracellular polymeric substances formation without compromising bacterial survival. Furthermore, smI‐loaded tFNA demonstrated a reduction in the severity of dental caries in vivo without adversely affecting oral microbial diversity and exhibited desirable topical and systemic biosafety. This study emphasizes the concept of ‘ecological prevention of biofilm’, which is anticipated to advance the optimization of biofilm prevention strategies and the clinical application of DNA nanocarrier‐based drug formulations.

## Graphical abstract

This study marks the first confirmation that tetrahedral framework nucleic acid efficiently loads the anti‐biofilm small‐molecule inhibitor, which inhibits biofilm formation in vitro and in vivo without bactericidal effects on pathogenic and symbiotic bacteria. The study introduces the innovative concept of ‘ecological prevention of biofilm’ for advancing the precise prevention of biofilm‐related diseases.

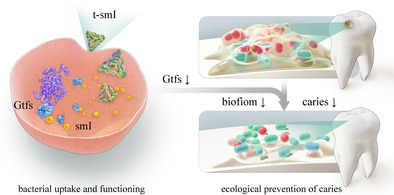

## 1 INTRODUCTION

Biofilm, referred to as a bacterial ‘city–state’, primarily consists of polysaccharides, nucleic acids and proteins secreted by bacteria.^1^, ^2^ Bacteria within biofilms exhibit increased resistance to antibiotics, ranging from tens to hundreds of times that of planktonic bacteria. In addition, they demonstrate closer intracellular communication, making it challenging to treat biofilm‐related diseases.^3^, ^4^, ^5^ The characteristics and prevention priorities of such diseases vary significantly depending on tissue environments or bacteria involved. Methicillin‐resistant *Staphylococcus aureus* is a major contributor to chronic wound infections, and wound healing necessitates the principle of ‘sterility’.^6^ Ideal drugs should possess both bactericidal and anti‐biofilm effects, exemplified by silver nanoparticles and antimicrobial peptides.^7^, ^8^ In contrast, various tissues and organs in the human body, such as the oral cavity, gastrointestinal tract and vagina,^9^, ^10^ host microecological systems. Microbial communities, including pathobionts, contribute to ecological balance and homeostasis, and participate in host immunity and metabolism. However, the bactericidal effect of drugs may disrupt microbial balance and impact host health.

Many conventional drugs, including antimicrobial peptides,^11^, ^12^, ^13^ indirectly exert anti‐biofilm effects through sterilization, with the effective concentration prioritizing bactericidal over anti‐biofilm effects.^14^ Small‐molecule inhibitors (smIs), synthesized artificially with ~1000 molecular weight and high affinity to specific targets,^15^, ^16^, ^17^ offer an alternative. For instance, a smI named 2‐(4‐methoxyphenyl)‐*N*‐(3‐{[2‐(4‐methoxyphenyl)ethyl]imino}‐1,4‐dihydro‐2‐quinoxalinylidene)ethanamine specifically inhibits intracellular glucosyltransferase C (GtfC) in *Streptococcus mutans*,^18^ the primary pathobiont responsible for cariogenic biofilm formation.^2^, ^19^, ^20^ Although smIs have the potential to efficiently inhibit extracellular polymeric substances (EPS) formation without affecting bacterial survival, their limited uptake efficiency necessitates suitable drug delivery carriers.

The tetrahedral framework nucleic acid (tFNA) stands out as a representative DNA nanomaterial with high programmability.^21^, ^22^, ^23^ Our previous studies indicate that tFNA boasts excellent bacterial/fungal uptake efficiency^24^, ^25^, ^26^ and can deliver various small‐molecule drugs, such as curcumin,^27^, ^28^ quercetin,^29^ glabridin,^30^ and wogonin.^31^ This study represents the first utilization of tFNA as a delivery vector for anti‐biofilm smIs, aiming to explore therapeutic effects through in vitro and in vivo caries models. The objective is to efficiently inhibit EPS formation while maintaining ecological balance, thereby contributing to the optimization of biofilm prevention strategies and the clinical transformation of DNA nanomaterial‐based drug formulations.

## 2 METHODS

### 2.1 Fabrication of tFNA and smI‐loaded tFNA

Four single‐stranded (ss)DNAs (sequences listed in Table S1) (Sangon Biotech, Shanghai, China) were purified by polyacrylamide‐gel electrophoresis (PAGE) for accurate quantification. The four ssDNAs, denoted as S1, S2, S3 and S4, were prepared with equimolar concentrations and simultaneously mixed in the TM buffer. The mixture was heated to 95°C for 10 min and cooled quickly to 4°C for 20 min; thus, the tFNA was synthesized. Subsequently, tFNA was incubated with the smI of GtfC, which is named 2‐(4‐methoxyphenyl)‐*N*‐(3‐{[2‐(4‐methoxyphenyl)ethyl]imino}‐1,4‐dihydro‐2‐quinoxalinylidene)ethanamine (Allkas, Beijing, China), at different molar ratios (1:100–1:500) at 4°C for 12 h; thus, the smI‐loaded tFNA (t‐smI) was synthesized.

### 2.2 Characterization of tFNA and t‐smI

To verify the predicted synthesis of tFNA, the nanomorphological feature and nanosize of the product (125 nM) were observed using transmission electron microscopy (TEM) (FEI, Hillsboro, USA) as well as atomic force microscopy (AFM) (Bruker Nano Inc., Billerica, USA). Meanwhile, 8% non‐denaturing PAGE was run in the Tris base+/acetic acid+/EDTA (TAE) buffer at 4°C to explore the relative molecular weights of each ssDNA as well as the combinations of two ssDNAs (S1 + S2), three ssDNAs (S1 + S2 + S3) and four ssDNAs (S1 + S2 + S3 + S4).

After t‐smI was synthesized, the hydrodynamic particle size (nm) and ζ potential (mV) of tFNA and t‐smI (1:100–1:500) (50 nM for both) were measured using a Zetasizer Nano ZS instrument (Malvern Instruments Ltd, Malvern, UK). The nanomorphological feature and nanosize of t‐smI (1:20, 1:100, 1:200, 1:300) were observed using TEM.

The loading efficiency of t‐smI was measured using a microplate reader (Thermo Fisher Scientific, USA). First, the absorbance of the smI solution (gradient concentration: 0, 20, 40, 60, 80, 100, 120 and 140 μg/mL) was recorded for obtaining the standard concentration–absorbance curve. Then, the t‐smI solution (1:100–1:500) was centrifuged at 6000 rpm for 5 min in a 3‐kD ultrafiltration tube (Ultra 30K device, Amicon) for separating loaded smI and unloaded smI. The concentration of unloaded smI within the sublayer solution was calculated according to the standard curve. The loading efficiency of t‐smI was determined according to the total and unloaded concentration of smI.

The ultraviolet–visible (UV–vis) profiles of tFNA and t‐smI (calibrated with the smI monomer) between 0 and 600 nm were explored to reveal the specific binding of t‐smI. Furthermore, the loading mechanism of t‐smI was investigated using a competitive‐binding test. Hoechst 33342 (MedChemExpress, Monmouth Junction, NJ, USA) was incubated with the t‐smI solution (1:100–1:500) at 4°C for 20 min, and then analysed at the wavelength of 461 nm using a microplate reader.

### 2.3 Bacterial culture and growth conditions

*S. mutans* UA159, *Streptococcus sanguinis* ATCC 10556 and *Streptococcus gordonii* ATCC 10558 were strains from our laboratory collection. All the bacteria strains were routinely grown in the brain heart infusion (BHI) medium (Solarbio, Beijing, China) within an anaerobic chamber (5% CO_2_, 10% H_2_ and 85% N_2_) at 37°C. For biofilm formation, 1% sucrose (Sangon Biotech, Shanghai, China) was added to BHI (BHIS).

The initial concentration of bacteria was adjusted to 1 × 10^4^ colony forming unit (CFU) (mL^−1^ in BHI for all the experiments unless noted otherwise. For multispecies culture and biofilm formation, the three bacteria strains were mixed at the ratio of 1:1:1 with the total concentration of 1 × 10^4^ CFU mL^−1^.

For full dissolving of smI, dimethyl sulfoxide (DMSO) (MedChemExpress, Monmouth Junction, NJ, USA) was used with the final concentration <1%. The control samples were also added with DMSO for desirable comparability.

For drug treatment, smI was used at 5 μg/mL, tFNA/smI was used at the molar ratio of 1:20, unless noted otherwise.

### 2.4 Drug uptake efficiency by *S. mutans*

Cyanine‐5 (Cy5) was linked to S1 for fluorescent labelling of tFNA and t‐smI. Cy5‐tFNA (500 nM) and Cy5‐t‐smI (500 nM) were, respectively, used to treat *S. mutans* grown in BHI for 6 and 12 h. Then, the samples were collected, resuspended thrice using PBS (5000 rpm, 5 min) (MedChemExpress, Monmouth Junction, NJ, USA) and finally subjected to flow cytometric analysis (FC500; Beckman, America).

### 2.5 Live/dead analysis of *S. mutans*

*S. mutans* (1 × 10^6^ CFU mL^−1^) cultured in BHI was subjected to different drug treatments for 1 h, respectively. Subsequently, the samples were collected, washed thrice using PBS (5000 rpm, 5 min), stained with SYTO9/Propidium Iodide (PI) (100:1) (Thermo Fisher Scientific, Shanghai, China) for 15 min, resuspended in PBS and finally observed under confocal laser scanning microscope (CLSM) (A1R MP+, Nikon, Japan).

*S. mutans* (1 × 10^6^ CFU mL^−1^) was treated in the same way except that only PI (100:1) was used for fluorescent labeling and subjected to flow cytometric analysis (*n* = 3).

### 2.6 Bacterial growth assay

*S. mutans* as well as a mixture of the three bacteria strains was grown in BHI in a 96‐well plate and was subjected to different drug treatments for 24 h, respectively. An automated spectrophotometer (BioTek, Winooski, USA) was used to record the optical density (OD) value at 595 nm every 4 h (*n* = 3), with the plate shaking every 30 min.

*S. mutans* as well as a mixture of the three bacteria strains was treated in the same way on a BHI agar plate, for intuitive observation of plate colony count.

### 2.7 Concentration–toxicity assessment of smI

The OD value at 600 nm of smI (0–200 μg/mL) was recorded. Then, smI of different concentrations was used to treat *S. mutans* as well as the multispecies in BHI for 24 h. The OD value at 600 nm of *S. mutans* as well as the multispecies was recorded and calibrated with the smI (*n* = 3), which reflected the inhibition of bacterial growth.

### 2.8 Microscopic observation of bacterial biofilm

*S. mutans* was subjected to different drug treatments in BHIS on a confocal plate for 24 h. Then, the medium was discarded and each sample was washed thrice using PBS. Then *S. mutans* was incubated with SYTO9 and TRITC‐ConA (Kaixin Biotech, Xi'an, China) for 15 min, for fluorescent labeling of bacteria (green) and extracellular polysaccharide (EPS) (orange), respectively. After PBS washing, the architecture of *S. mutans* biofilm was observed using CLSM (A1R MP+; Nikon, Tokyo, Japan), and the three‐dimensional images were reconstructed. The COMSTAT image processing software was used for analysing the coverage of bacteria/EPS within the 30‐μm depth.

Moreover, *S. mutans* was subjected to different drug treatments in BHIS on a 24‐well plate for 24 h. The biofilm was rinsed with PBS, fixed in 2.5% glutaraldehyde (MedChemExpress, Monmouth Junction, NJ, USA) overnight and serially dehydrated using ethanol (Sangon Biotech, Shanghai, China) for 30 min (40%, 60%, 80%, 100%). Finally each sample was observed using a scanning electron microscope (Inspect F; FEI, the Netherlands).

### 2.9 Quantitative analysis of biofilm formation

*S. mutans* cultured on a 96‐well plate containing BHIS was subjected to different drug treatments for 24 h. The medium was removed, and each sample was washed thrice with PBS. Each sample was fixed by 100 μL methanol (Sangon Biotech, Shanghai, China) for 15 min and dried naturally. Subsequently, each sample was incubated with 0.1% crystal violet staining solution (MedChemExpress, Monmouth Junction, NJ, USA) and placed at 20°C for 5 min. After each sample was washed with PBS, the plate was fully dried in a drying oven. Finally, the OD value at 595 nm of each sample was measured with a microplate reader (*n* = 3), and representative images of crystal violet staining were recorded.

The water‐insoluble EPS formation was analysed using the anthrone method. The biofilm was collected using PBS and centrifugated (6000 rpm, 5 min), and then the precipitates were resuspended in 0.4 M NaOH (Sangon Biotech, Shanghai, China). The supernatant (200 μL) was mixed with the anthrone reagent (600 μL) (MedChemExpress, Monmouth Junction, NJ, USA) at 95°C for 6 min. Finally, the absorbance at 625 nm was detected to reflect the water‐insoluble EPS formation (*n* = 3).

### 2.10 Gene expression analysis

Quantitative reverse transcription‐polymerase chain reaction (RT‐PCR) was used to measure the expression levels of biofilm‐related genes (*n* = 3). 16S rRNA was used as a control gene. *S. mutans* was grown in BHIS and subjected to different drug treatments for 24 h. Next, each sample was centrifugated (4000*g*, 4°C, 10 min) and frozen in TRIzol reagent (Thermo Fisher Scientific, USA). Total RNA was carefully extracted and purified using the RNeasy Mini Kit (Qiagen, Hilden, Germany), and the extracted samples were dissolved in RNase‐free water (Thermo Fisher Scientific, USA). A cDNA synthesis kit (Thermo Fisher Scientific, USA) was used for cDNA preparation. Amplification of all target mRNAs (*gtfB*, *gtfC*, *gtfD*, *ftf*, *vicR*, *vicK*, *comC* and *comD*) was performed based on corresponding primer sets listed in Table S2. Especially, the control group and smI groups also contained equimolar tFNA to the tFNA and t‐smI group for reduced bias.

### 2.11 Establishment of an in vivo Caries Model

All the in vivo experiments were authorized by the Medical Ethics Committee of the Hospital of Stomatology, Sun Yat‐Sen University. Twenty‐five female specific‐pathogen‐free Sprague–Dawley rats aged 17 days (weight 45 ± 5 g) were purchased from Dossy, Chengdu, China. Five rats were randomly chosen as the healthy group that were routinely fed without caries induction. The remaining rats were fed with distilled water containing 4000 U/mL penicillin (Sangon Biotech, Shanghai, China) and standard laboratory chow for 3 days. Then, the tooth surfaces of rats were infected with *S. mutans* (1 × 10^6^ CFU mL^−1^) for 5 min once daily for 3 days. The infected rats were randomly divided into four groups: (1) untreated (control), (2) treated with tFNA, (3) treated with smI and (4) treated with t‐smI. The rats were treated using a camel hair brush pre‐immersed in 2 mL drug solutions, for 5 min thrice daily (morning, afternoon and evening) for 5 weeks. For the experimental groups, smI was used at 5 μg/mL and tFNA/smI was used at the molar ratio of 1:20. The Bass brushing action was softly performed towards each surface of each tooth. Meanwhile, the rats were fed with the cariogenic Keyes 2000 diet and 5% sucrose water during the 5‐week treatment period.

### 2.12 Keyes scoring

After the 5‐week treatment period, all the rats were sacrificed by CO_2_ asphyxiation. The jaws were collected and stained with 0.4% murexide (Chron Chemicals, Chengdu, China) for 12 h. The main sulcus of each tooth was observed through a mesiodistal sagittal hemisection. The sulcus surfaces and smooth surfaces were both analysed in terms of caries severity using the Keyes scoring system (*n* = 5).

### 2.13 Microbiota analysis in vivo

Immediately after the 5‐week treatment, the saliva was sampled from each rat, and then genomic DNA was extracted and purified using a QIAamp DNA minikit (Qiagen, Hilden, Germany). The resultant DNA samples were subjected to 16S rRNA gene sequencing. For α‐diversity, the Shannon index and the Chao index were explored at the operational taxonomic unit (OTU) level. For β‐diversity, Jaccard‐based principal coordinates analysis (PCoA) was investigated at the OTU level.

### 2.14 Biosafety assessment in vivo

The body mass of each rat was recorded every week since drug treatment (*n* = 5).

After sacrifice, the gingival crevicular fluid was collected and subjected to enzyme‐linked immunosorbent assay (ELISA) concerning tumor necrosis factor‐α (TNF‐α), interleukin‐6(IL‐6) and interleukin‐1β (IL‐1β) (Beyotime, Shanghai, China), to reflect the topical inflammatory level (*n* = 3). Furthermore, the oral mucosa, heart, spleen and kidney were carefully dissected and subjected to haematoxylin–eosin (H&E) staining for topical and systemic biosafety assessment (*n* = 3).

### 2.15 Statistical analysis

All the experiments were conducted at least three separate times for desirable reproducibility, and all the quantitative data were presented as the mean value ± standard deviation. All the statistical description and analysis were performed based on GraphPad Prism (version 8.0.2, San Diego, USA) and one‐way analysis of variance followed by Tukey's post hoc test. A *p*‐value less than 0.05 indicated a statistically significant difference.

## 3 RESULTS AND DISCUSSION

### 3.1 Characterization and bacterial uptake assessment of t‐smI

**FIGURE 1:**
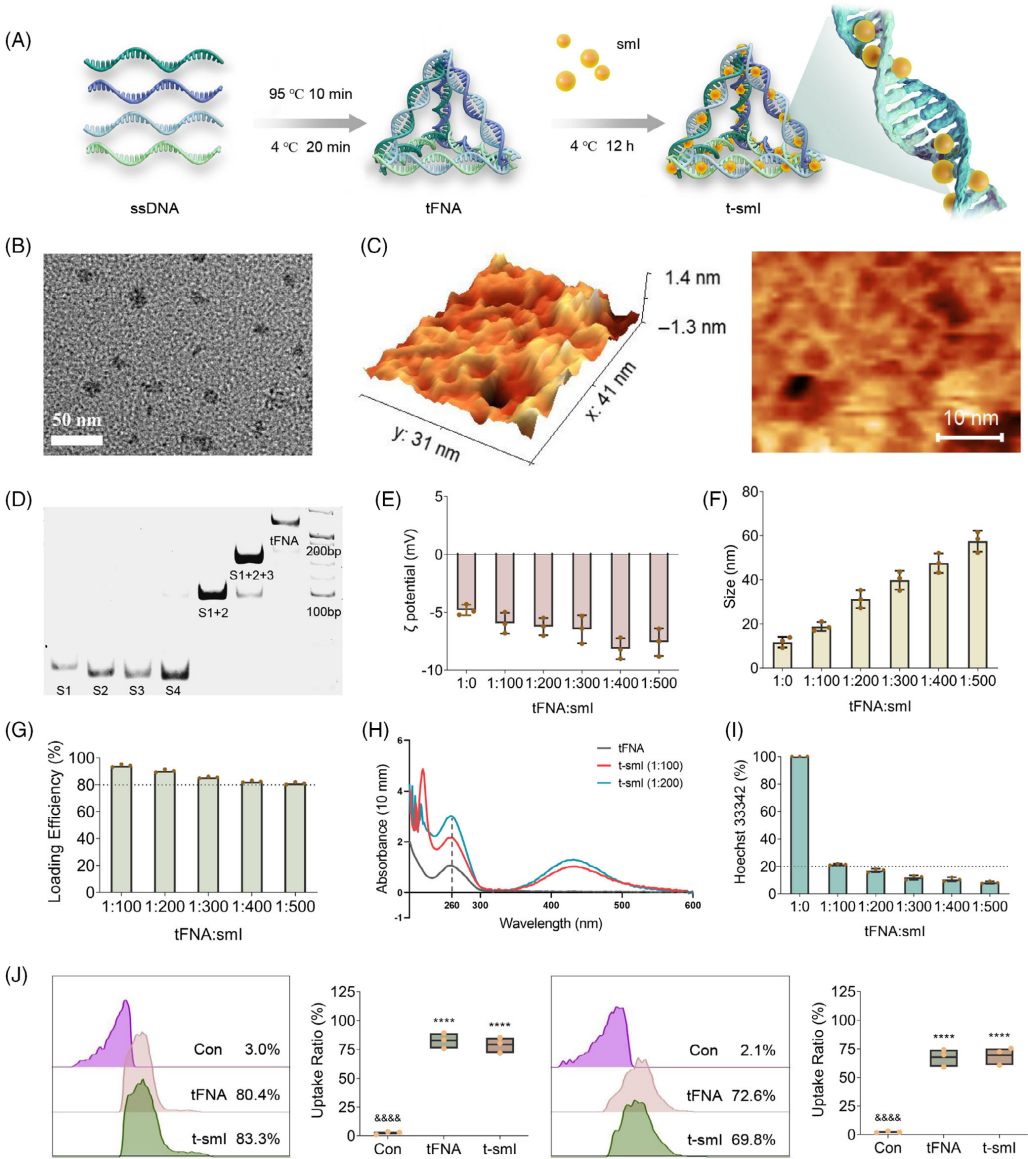
Synthesis and characterization of materials. (A) Schematic diagram illustrating the synthesis process of tFNA and t‐smI. (B) TEM observation of tFNA. Scale bar = 50 nm. (C) AFM observation of tFNA. Scale bar = 10 nm. (D) Molecular weight analysis of ssDNAs and tFNA using 8% PAGE. (E) ζ potential analysis of tFNA and t‐smI. (F) Particle size analysis of tFNA and t‐smI. (G) Loading efficiency analysis of t‐smI. (H) UV–vis spectrum of tFNA and t‐smI. (I) Competitive binding test of Hoechst 33342 with t‐smI. (J) Uptake efficiency of tFNA and t‐smI towards *Streptococcus mutans* assessed by flow cytometry. Compared with the Con group, ****p < 0.0001; compared with the t‐smI group, ^&&&^ *p* < 0.0001. AFM, atomic force microscopy; PAGE, polyacrylamide‐gel electrophoresis; ssDNAs, single‐stranded DNAs; TEM, transmission electron microscopy; tFNA, tetrahedral framework nucleic acid; t‐smI, small‐molecule inhibitor‐loaded tFNA; UV–vis, ultraviolet–visible.

The schematic diagram illustrating the synthesis of tFNA and t‐smI is presented in Figure 1a, with the chemical structure of smI depicted in Figure S1. Initially, a one‐pot annealing method (95°C for 10 min, 4°C for 20 min) was employed to synthesize tFNA using four ssDNAs (sequences provided in Table S1). Each ssDNA, comprising 63 bases, is paired with the other three single strands to form three edges and one of the four faces of tFNA. The resulting product exhibited a framework structure under TEM (Figure 1b) and AFM (Figure 1c). PAGE revealed a gradual increase in molecular weight compared to ssDNAs and partially self‐assembled products (Figure 1d).

Subsequently, tFNA and smI were incubated at various ratios (1:100–1:500) at 4°C for 12 h to generate t‐smI. According to the laser particle size analyser, with an increase in the loading amount of smI, the ζ potential of t‐smI ranged between −5 and −10 mV (Figure 1e), and the particle size of t‐smI increased from ~10 to ~60 nm (Figure 1f). Based on the concentration–absorbance curve of smI determined by spectrophotometry (Figure S2), the binding efficiency of t‐smI exceeded 80% under the selected loading ratios (1:100–1:500) as determined by ultrafiltration (Figure 1g). This efficiency surpassed that of tFNA binding with traditional small‐molecule drugs such as paclitaxel.^32^, ^33^

In terms of the UV–vis spectrum (Figure 1h), the absorbance at 260 nm (the characteristic peak of DNA) gradually increases as smI is loaded from 1:100 to 1:200 with the absorbance of smI calibrated, which confirms the structural change of DNA and successful loading of smI. A competitive binding assay was performed to validate the binding site of smI (Figure 1i). The binding efficiency of Hoechst 33342 with t‐smI (1:0–1:500) gradually decreased from 100% to ~10%, indicating that smI competed with Hoechst 33342 for the small grooves of the DNA double helix. In subsequent experiments, the ratio of t‐smI was determined to be 1:20, taking into account the biosafe concentration of tFNA, the effective concentration of smI and the minimal particle size increase at this ratio (TEM images shown in Figure S3).

Cy5 was utilized for fluorescence labeling of tFNA to assess the uptake efficiency of tFNA and t‐smI (both at 500 nM) in terms of *S. mutans* through flow cytometry (Figure 1j). After 6‐h treatment, the uptake efficiency of both tFNA and t‐smI exceeded 80%, while after 12‐h treatment, the uptake efficiency for both was close to 70%. In line with literature findings,^25^ tFNA demonstrated excellent uptake by *S. mutans* even after smI loading, establishing it as a suitable delivery carrier.

### 3.2 Effect of t‐smI on the survival of *S. mutans* and multiple bacterial strains

The fundamental premise of this study is that the developed anti‐biofilm drug formulation should exert minimal inhibitory effects on bacterial survival, particularly for pathogenic bacteria. According to prior literature,^18^ the effective concentration of smI in this study was determined to be 5 μg/mL, both in vitro and in vivo.

**FIGURE 2:**
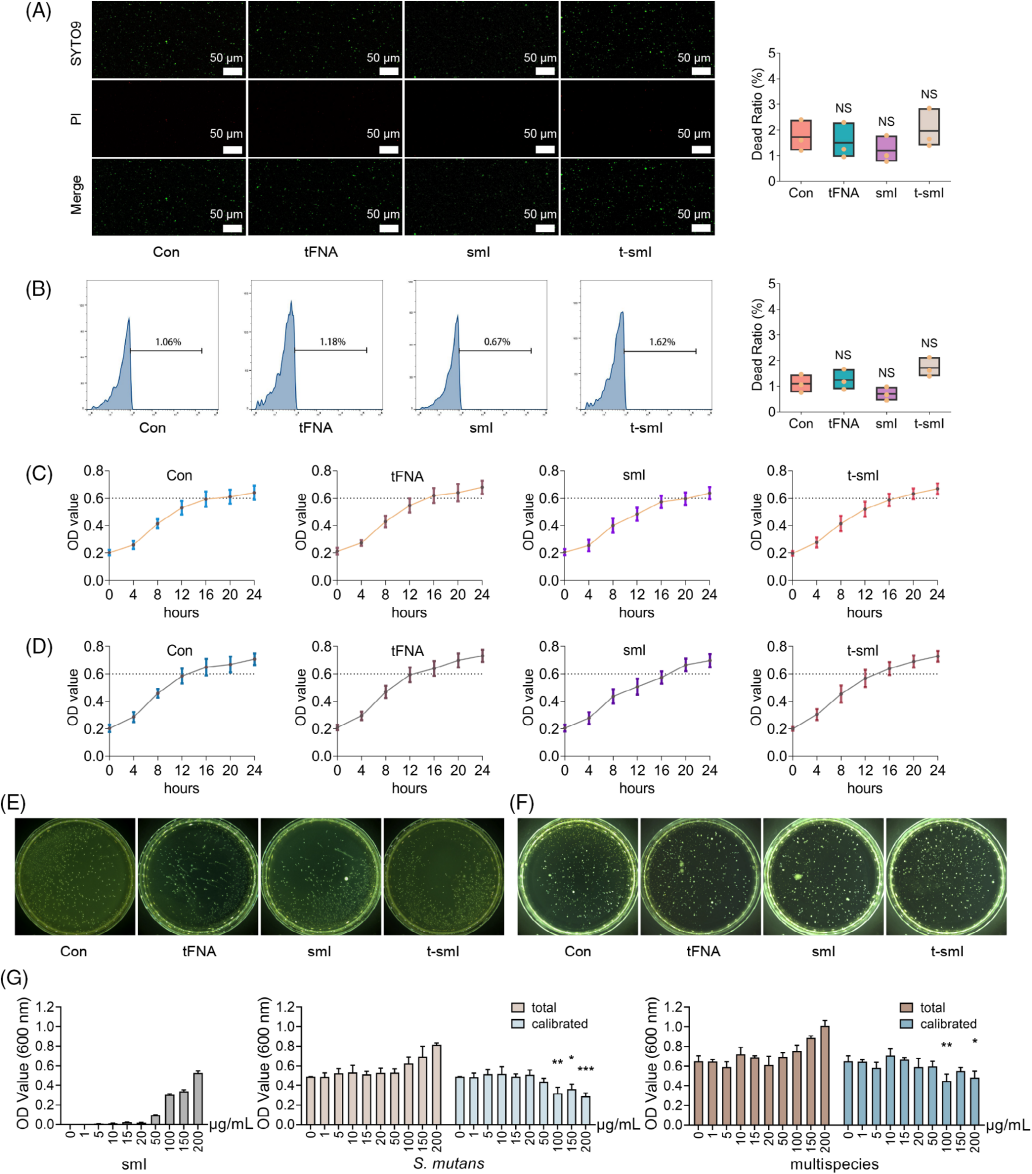
Determination of bacterial survival after drug treatment. (A) SYTO9/PI confocal observation of the live/dead ratio of *Streptococcus mutans*. (B) Flow cytometry for assessing the live/dead ratio of *S. mutans*. NS indicates no statistical difference. (C, D) The 24‐h growth curves of single and multiple bacterial strains after drug treatment, respectively. (E, F) The 24‐h plate counting observation of single and multiple bacterial strains after drug treatment, respectively. (G) OD values of gradient concentration of smI and 24‐h growth of single and multiple bacterial strains after smI treatment. Compared with the 0 nM group, *p < 0.05, **p < 0.01, ***p < 0.001. smI, small‐molecule inhibitor.

Initially, the immediate bactericidal effect was assessed by subjecting *S. mutans* to various drug treatments for 1 h. SYTO9/PI staining of live and dead cells (Figure 2a) indicated that the drug treatment scarcely induced bacterial death, with the proportion of dead bacteria in each group being less than 2%. Flow cytometry analysis of the same process corroborated these findings (Figure 2b), revealing that the bacterial mortality rate in each group was below 2%. In summary, t‐smI exhibited almost no immediate bactericidal effect.

The 24‐h growth curve of bacteria post‐drug treatment was then examined. For both the single strain of *S. mutans* (Figure 2c) and multiple strains, including *S. mutans*, *S. gordonii* and *Streptococcus sanguis* (Figure 2d), the 24‐h growth curves in each group were essentially consistent. Similarly, plate counting experiments (Figure 2e for single strain and Figure 2f for multiple strains) yielded predominantly uniform results. Collectively, t‐smI demonstrated minimal inhibition of bacterial survival.

We further investigated whether smI exhibited any anti‐bacterial effect at higher concentrations. Bacterial growth after 24‐h treatment with varying concentrations of smI was recorded (Figure 2g). An anti‐bacterial effect on *S. mutans* and multiple bacterial strains was observed only when the concentration of smI exceeded 100 μg/mL. The concentration utilized in this study (5 μg/mL) was significantly lower than 100 μg/mL, thereby ensuring efficient biofilm inhibition without adversely affecting bacterial survival.

### 3.3 Preventive effect of t‐smI on EPS formation of *S. mutans*

**FIGURE 3:**
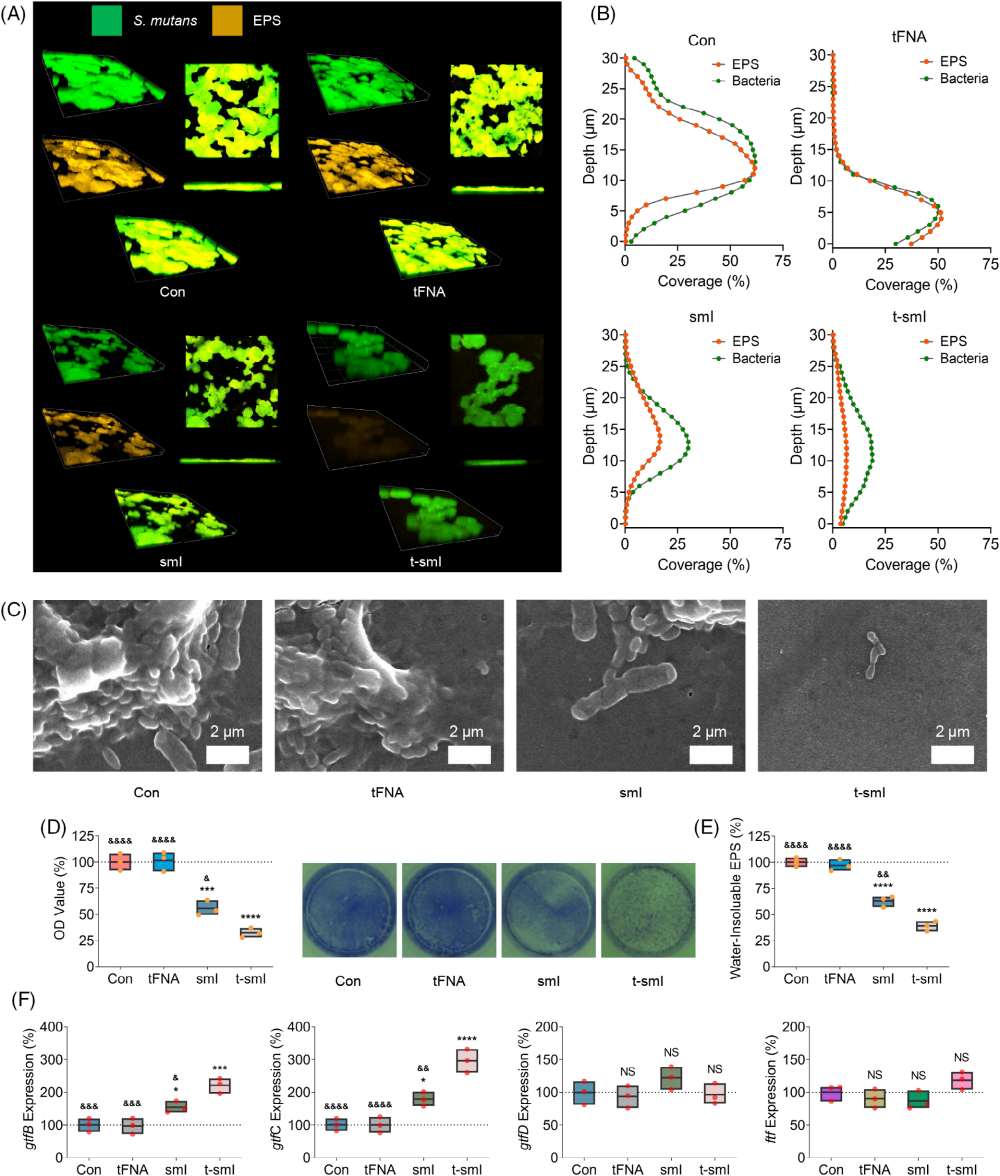
Analysis of biofilm formation and related genes in *Streptococcus mutans* after drug treatment. (A) SYTO9/TRITC ConA confocal observation of EPS generation. (B) Vertical distribution of bacteria and EPS from the surface to the bottom of the biofilm. (C) Observation of biofilm formation and the morphology of *S. mutans* using SEM. Scale bar = 2 μm. (D) Observation and quantitative analysis of crystal violet staining. (E) Quantitative analysis of water‐insoluble EPS production using the anthrone method. (F) Quantitative analysis of gene expression related to biofilm formation using qPCR. Compared with the Con group, *p < 0.05, ***p < 0.001, ****p < 0.0001; compared with the t‐smI group, ^&^ *p* < 0.05, ^&&^ *p* < 0.01, ^&&&^ *p* < 0.001, ^&&&&^ *p* < 0.0001; NS indicates no statistical difference. EPS, extracellular polymeric substances; qPCR, quantitative polymerase chain reaction; SEM, scanning electron microscopy; t‐smI, t‐smI, small‐molecule inhibitor‐loaded tetrahedral framework nucleic acid.

Initially, the visualization of EPS formation by *S. mutans* was conducted using confocal microscopy (Figure 3a) with SYTO9/TRITC ConA staining (green: bacteria, orange: EPS). The images clearly revealed that tFNA alone had minimal impact on EPS formation, whereas smI and t‐smI successively inhibited EPS formation, as evidenced by a reduction in orange fluorescence. Quantitative assessment of the vertical distribution of bacteria and EPS within the biofilm (Figure 3b) confirmed a significant decrease in the EPS/bacterial ratio with t‐smI. Scanning electron microscopy (SEM) observations (Figure S4 large view and Figure 3c small view) further supported these findings, demonstrating a sequential reduction in biofilm structure from the control group to the t‐smI group, while the cellular integrity was hardly destructed.

Crystal violet staining and quantitative analysis (Figure 3d) indicated that EPS formation in the t‐smI group decreased to ~30% of the control group. In addition, the anthrone method was employed for quantitative measurement of EPS formation (Figure 3e), revealing a reduction in water‐insoluble EPS formation in the t‐smI group to ~40% of the control group.

Analysis of gene expression related to EPS formation was carried out using quantitative PCR (qPCR) (Figure 3f and primer sequences provided in Table S2). The expression of *gtfC* increased sequentially in the smI and t‐smI groups. As GtfC is the target of smI, the elevation in *gtfC* gene expression may be attributed to a compensatory effect due to inhibited GtfC activity and EPS formation. Simultaneously, the expression of *gtfB* increased sequentially in the smI and t‐smI groups, while the expression of *gtfD* and *ftf* exhibited no significant differences among the groups. This may be explained by the higher homology of *gtfB* and *gtfC*.^2^ Furthermore, gene expression associated with the dual‐component signal transduction system was analysed (Figure S5 and primer sequences provided in Table S2). The expression of *comC* and *comD* decreased sequentially in the smI and t‐smI groups, while the expression of *vicK* and *vicR* showed no significant differences in each group, indicating a partial inhibition of the dual‐component signal transduction system.

### 3.4 Ecological prevention of biofilm by t‐smI in a rat caries model

**FIGURE 4:**
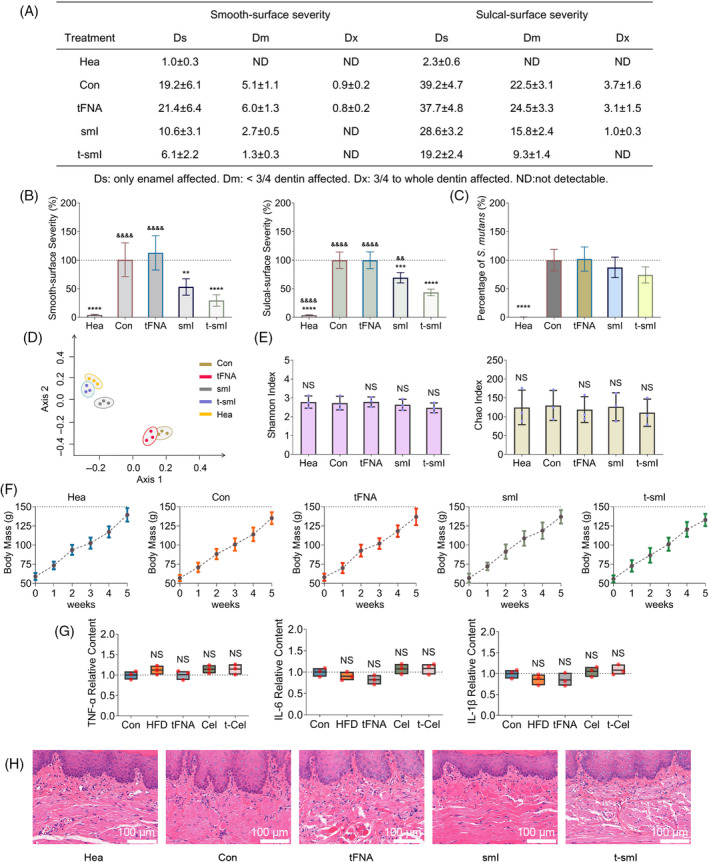
Ecological prevention of biofilm in a rat caries model. (A) Keyes scoring results of rat caries. (B) Quantitative analysis of the severity of dental caries. (C) Relative content of *Streptococcus mutans* in each group. (D) PCoA analysis of microbial composition similarity. (e) α‐diversity analysis of microbial richness. (f) Changes in rat weight within 5 weeks. (g) Quantitative analysis of inflammatory cytokines in the gingival crevicular fluid using ELISA. (h) H&E histological observation of oral mucosa. Scale bar = 100 nm. Compared with the Con group, **p < 0.01, ***p < 0.001, ***p < 0.0001; compared with the t‐smI group, ^&&^ *p* < 0.01, ^&&&&^ *p* < 0.0001; NS indicates no statistical difference. ELISA, enzyme‐linked immunosorbent assay; PCoA, principal coordinates analysis.

A rat caries model was established and underwent programmatic drug administration.^12^ Keyes scoring was used at the final time point to assess caries severity (Figure 4a). The smooth‐surface caries and sulcal‐surface caries were measured separately and categorized into four levels based on caries severity (Ds, Dm, Dx, ND). Quantitative analysis (Figure 4b) revealed a sequential decrease in the severity of smooth‐surface caries in the smI and t‐smI groups, with the t‐smI group exhibiting a reduction to ~30% of the control group. Similarly, the severity of sulcal‐surface caries in the smI and t‐smI groups decreased sequentially, reaching ~40% of the control group in the t‐smI group. Moreover, 16S rRNA sequencing (Figure 4c) showed no statistically significant difference in the content of *S. mutans* among the groups. Thus, t‐smI demonstrated an anticaries effect by inhibiting the cariogenic virulence of *S. mutans* rather than reducing its content.

The composition and diversity of the oral microbiota were further examined. PCoA revealed that the control group and tFNA group shared a similar composition of oral microbiota, while the healthy group and t‐smI group shared a similar composition (Figure 4d). This suggested that the inhibitory effect of t‐smI on caries development contributed to the normalization of the entire species composition. In addition, there was no significant change in the diversity of the oral microbiota, as indicated by the Shannon index and the Chao index (Figure 4e), affirming that drug treatment maintained oral microbial homeostasis.

Finally, the biosafety of drug treatment was assessed. The weight change within the treatment period was consistent across groups (Figure 4f). Analysis of inflammatory cytokines in the gingival crevicular fluid via ELISA demonstrated consistent levels of TNF‐α, IL‐6 and IL‐1β in each group (Figure 4g). H&E staining revealed no significant pathological changes in the oral mucosa of each group (Figure 4h). In addition, outside the oral cavity, no significant pathological changes were observed in the heart, kidney and spleen of each group (Figure S6), indicating the desirable systemic biosafety of t‐smI.

## 4 CONCLUSION

**SCHEME 1:**
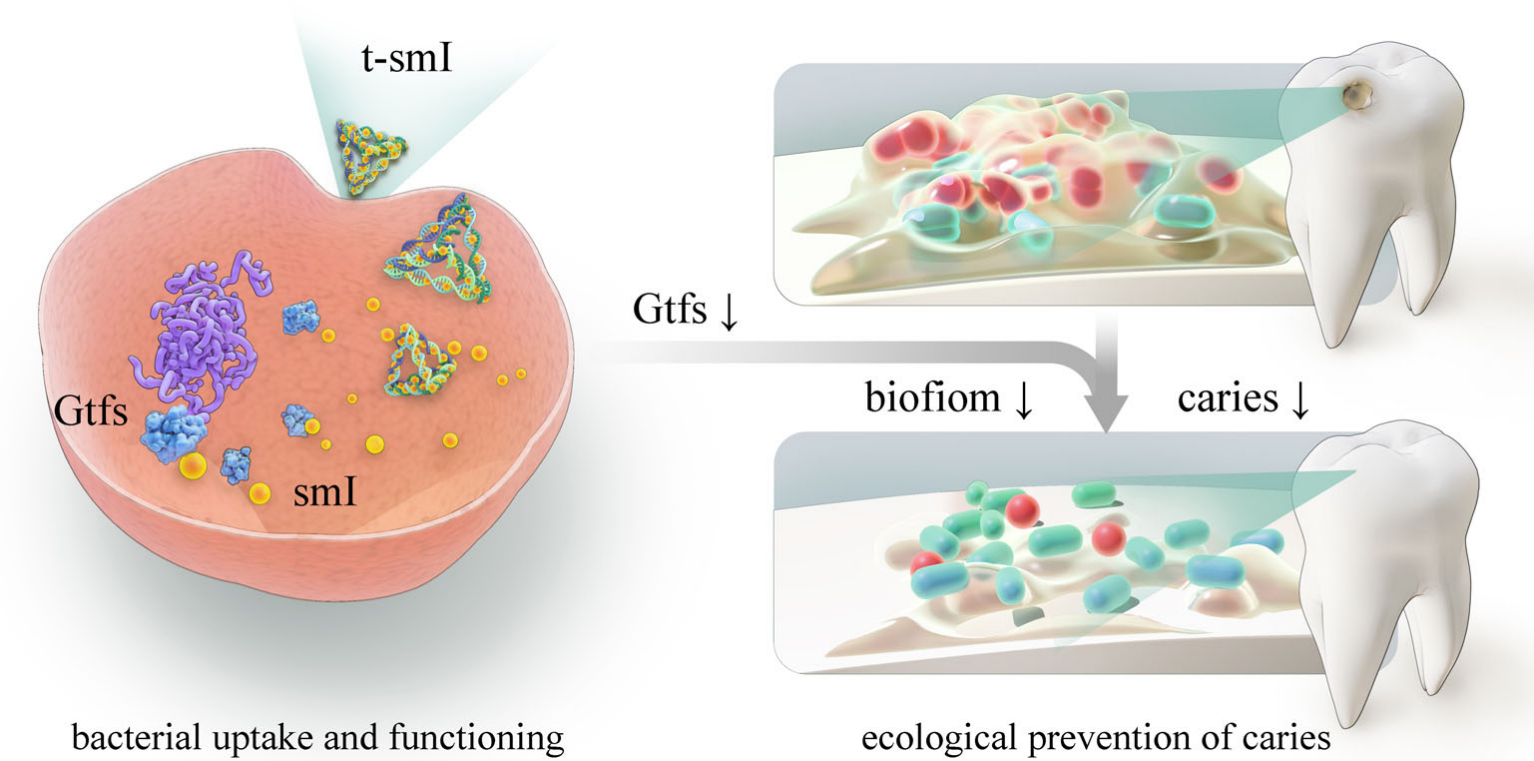
Ecological prevention of biofilm in vitro and in vivo by t‐smI. t‐smI, t‐smI, small‐molecule inhibitor‐loaded tetrahedral framework nucleic acid.

This study marks the first confirmation that tFNA efficiently loads the anti‐biofilm agent smI, enabling intracellular delivery to *S. mutans* without significantly compromising bacterial survival. Utilizing both in vitro and in vivo caries models, t‐smI proves effective in inhibiting EPS formation while avoiding bactericidal effects on both pathogenic and symbiotic bacteria. Furthermore, it demonstrates the capacity to hinder caries development while preserving microbial diversity and exhibiting both topical and systemic biosafety (Scheme 1). This delivery system is expected to simultaneously regulate Gtf activity and *gtf* expression in further studies, by co‐delivering smIs and other agents such as antisense oligonucleotides. Taken together, this study introduces the innovative concept of ‘ecological prevention of biofilm’, which integrates the anti‐biofilm efficacy with the maintenance of host microbial homeostasis. This approach holds promise for advancing the precise prevention and treatment of biofilm‐related diseases in tissues and organs characterized by microbial systems, such as the oral cavity, gastrointestinal tract and vagina.

## AUTHOR CONTRIBUTIONS

**Yuhao Liu:** Conceptualization; investigation; methodology; visualization; formal analysis; writing—original draft. **Kechen Li and Weijie Zhuang:** Methodology; formal analysis; visualization; software; resources. **Lulu Liang and Xiangyi Chen:** Data curation; software; project administration; validation. **Dongsheng Yu:** Supervision; resources; funding acquisition; writing—review and editing. Yuhao Liu, Kechen Li and Weijie Zhuang contributed equally to this work.

## FUNDING INFORMATION

This study received support from the National Natural Science Foundation of China (#82373255), China Postdoctoral Science Foundation (#2022TQ0381 and #2023M744045), and College Students' Innovative Entrepreneurial Training Plan Program (#20240504 and #20240516).

## CONFLICT OF INTEREST STATEMENT

The authors declare no competing financial interest.

## Supporting information


**Data S1.** Supporting Information.

## Data Availability

The data that support the findings of this study are available from the corresponding author upon reasonable request.
