# Supplementary material for: Tetrahedral framework nucleic acid–based small‐molecule inhibitor delivery for ecological prevention of biofilm
Source: Cell Prolif. 2024 May 29;57(9):e13678. doi: 10.1111/cpr.13678 (PMC11503243; doi:10.1111/cpr.13678)
Supplement: Supplementary file 1 — Data S1. Supporting Information. [file CPR-57-e13678-s001.docx]

**Tetrahedral Framework Nucleic Acid-based Small-Molecule Inhibitor Delivery for Ecological Prevention of Biofilm**

Yuhao Liu^a,1^, Kechen Li^a,1^, Weijie Zhuang^a,1^, Lulu Liang^a,b^, Xiangyi Chen^a^, Dongsheng Yu^a,*^

^a^Hospital of Stomatology, Guangdong Provincial Clinical Research Center of Oral Diseases, Guangdong Provincial Key Laboratory of Stomatology, Guangdong Key Laboratory for Dental Disease Prevention and Control, Sun Yat-Sen University, Guangzhou 510030, China

^b^Guangzhou Development District Hospital, Chinese Association of Medicinal Biotechnology, Southern Center of Biology Diagnosis and Therapy, Guangzhou 510730, China

^*^ Corresponding author.

E-mail address:[yudsh@mail.sysu.edu.cn](mailto:yudsh@mail.sysu.edu.cn)

^1^These authors contributed equally to this work.

**Table S1.** Sequences of the ssDNAs used in this study.

| ssDNA | Sequence (from 5′ to 3′ terminal) |
| --- | --- |
| S1 | ACGGTATTGGACCCTCGCATGACTCAACTGCCTGGTGATACGAGGATGGGCATGCTCTTCCCG |
| Cy5-S1 | Cy5-ACGGTATTGGACCCTCGCATGACTCAACTGCCTGGTGATACGAGGATGGGCATGCTCTTCCCG |
| S2 | ACTACTATGGCGGGTGATAAAACGTGTAGCAAGCTGTAATCGACGGGAAGAGCATGCCCATCC |
| S3 | ACATGCGAGGGTCCAATACCGACGATTACAGCTTGCTACACGATTCAGACTTAGGAATGTTCG |
| S4 | ATTTATCACCCGCCATAGTAGACGTATCACCAGGCAGTTGAGACGAACATTCCTAAGTCTGAA |

**Table S2.** Primers of target genes.

| Target gene | Sequence (from 5′ to 3′ terminal) |
| --- | --- |
| *gtfB*-F | ACGAACTTTGCCGTTATTGTCA |
| *gtfB*-R | AGCAATGCAGCCAATCTACAA |
| *gtfC*-F | CTCAACCAACCGCCACTGTT |
| *gtfC*-R | GGTTTAACGTCAAAATTAGCTGTATTAG |
| *gtfD*-F | TGTCTTGGTGGCCAGATAAAC |
| *gtfD*-R | GAACGGTTTGTGCAGCAAGG |
| *ftf*-F | CCTGCGACTTCATTACGATTGGTC |
| *ftf*-R | ATTGGCGAACGGCGACTTACTC |
| *comC*-F | TATCATTGGCGGAAGCGGAA |
| *comC*-R | TCCCCAAAGCTTGTGTAAAACT |
| *comD*-F | CGCGATTGGAGCCTTTAG |
| *comD*-R | CCTGAAATTCAGTTAGCCTTT |
| *vicK*-F | CACTTTACGCATTCGTTTTGCC |
| *vicK*-R | CGTTCTTCTTTTTCCTGTTCGGTC |
| *vicR*-F | CGCAGTGGCTGAGGAAAATG |
| *vicR*-R | ACCTGTGTGTGTCGCTAAGTGATG |


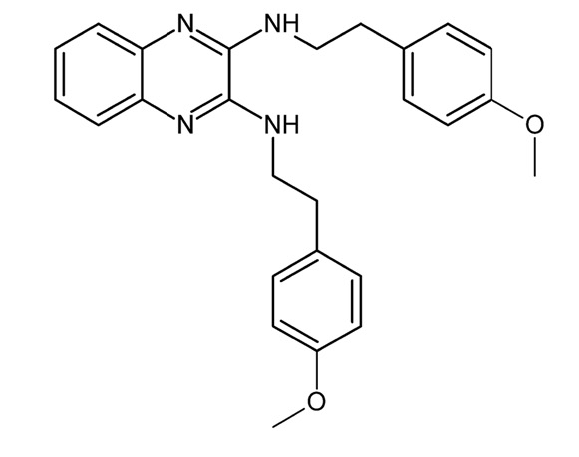


**Fig. S1.** The chemical structure of smI used in this study.


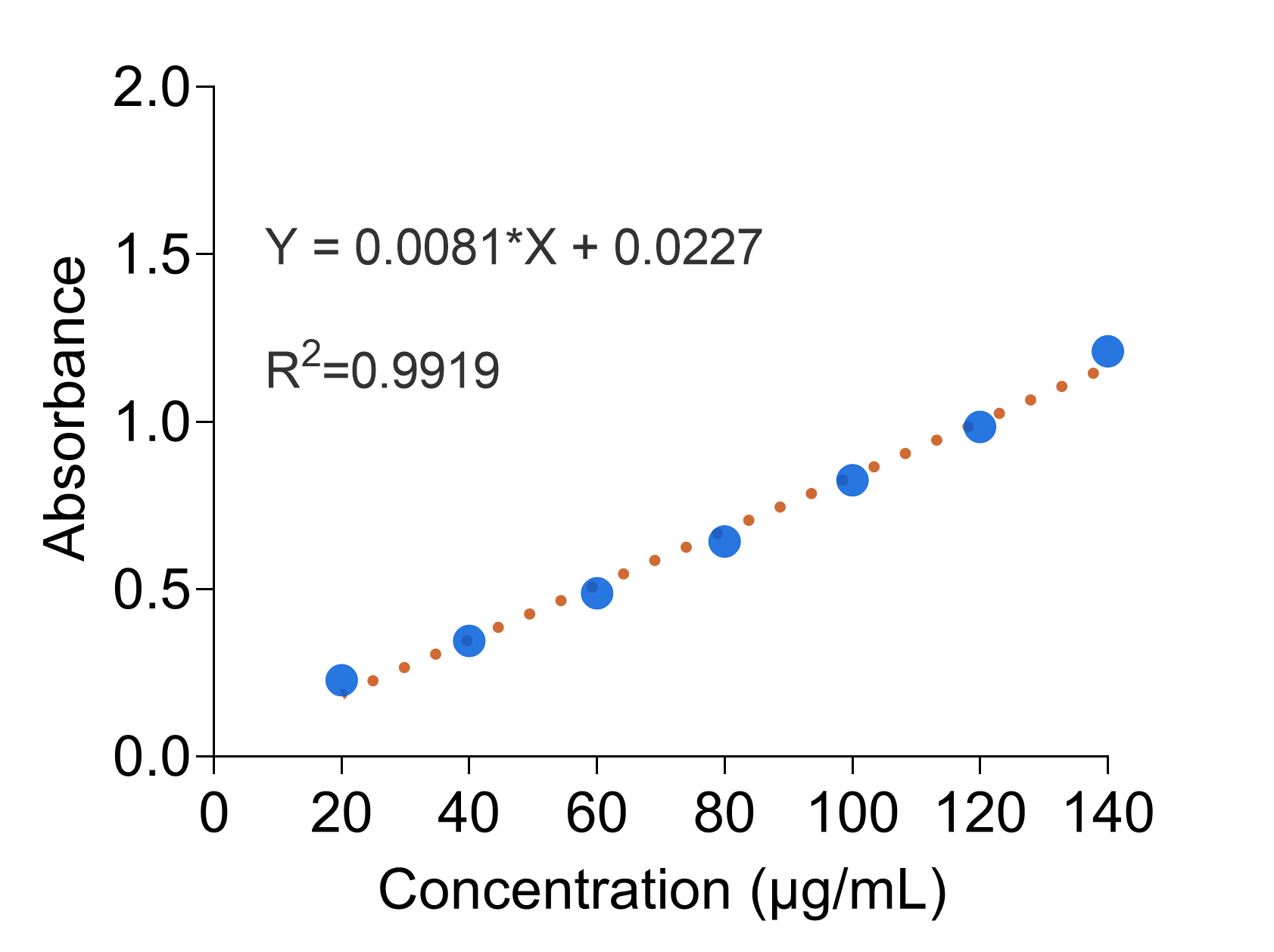


**Fig. S2.** The concentration-absorbance standard curve of smI.







t-smI (1:20) t-smI (1:100)







t-smI (1:200) t-smI (1:300)

**Fig. S3.** TEM observation of t-smI. Scale bar = 20 nm.


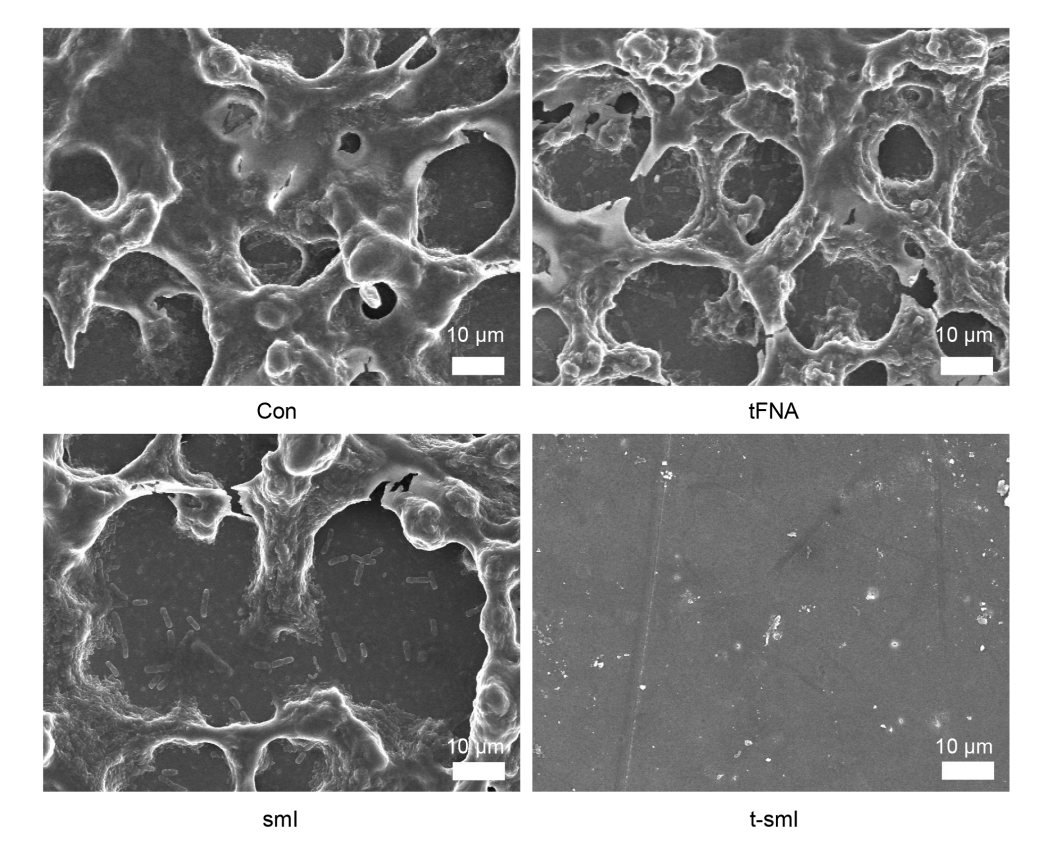


**Fig. S4.** SEM observation (large view). Scale bar = 10 μm.


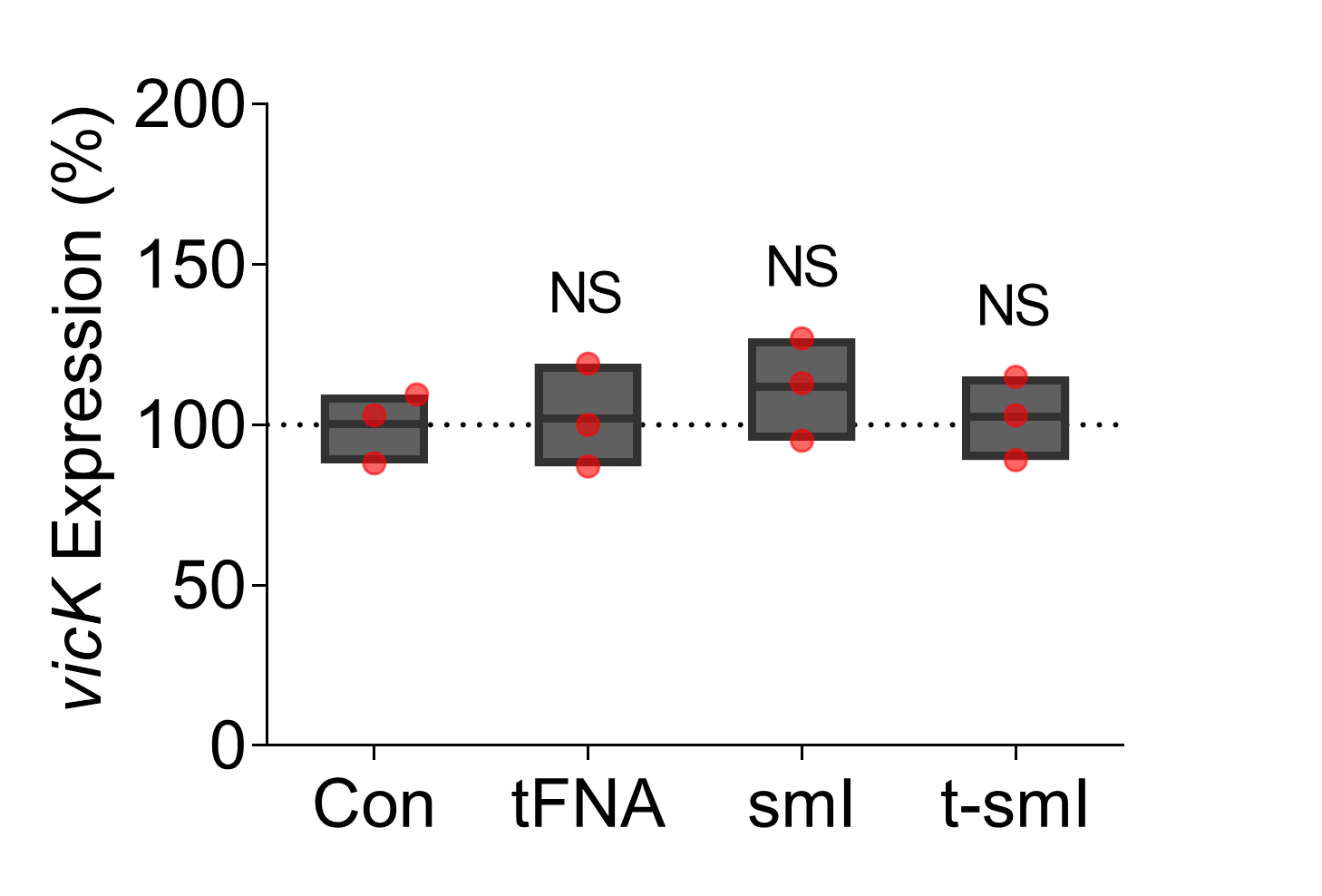

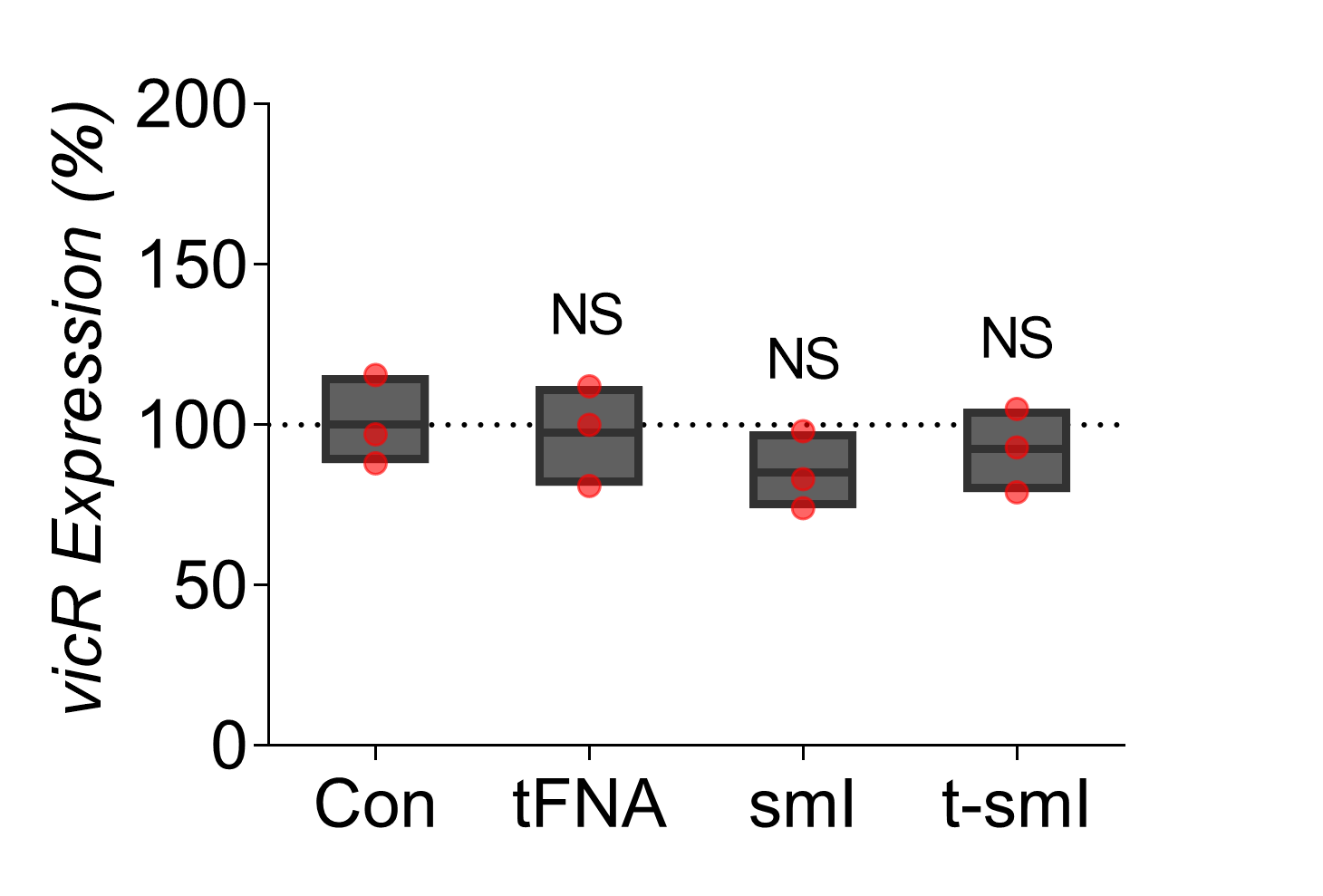


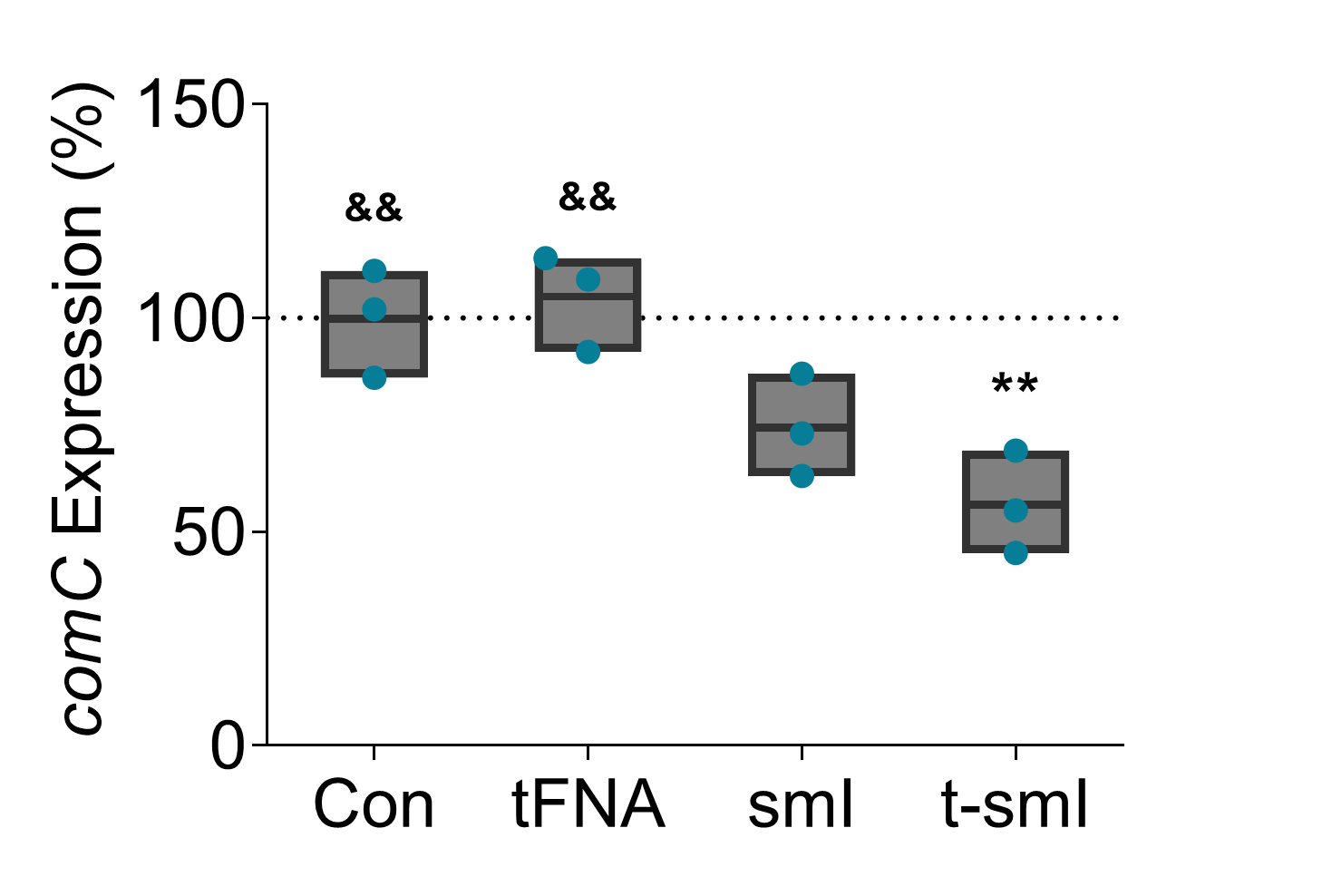

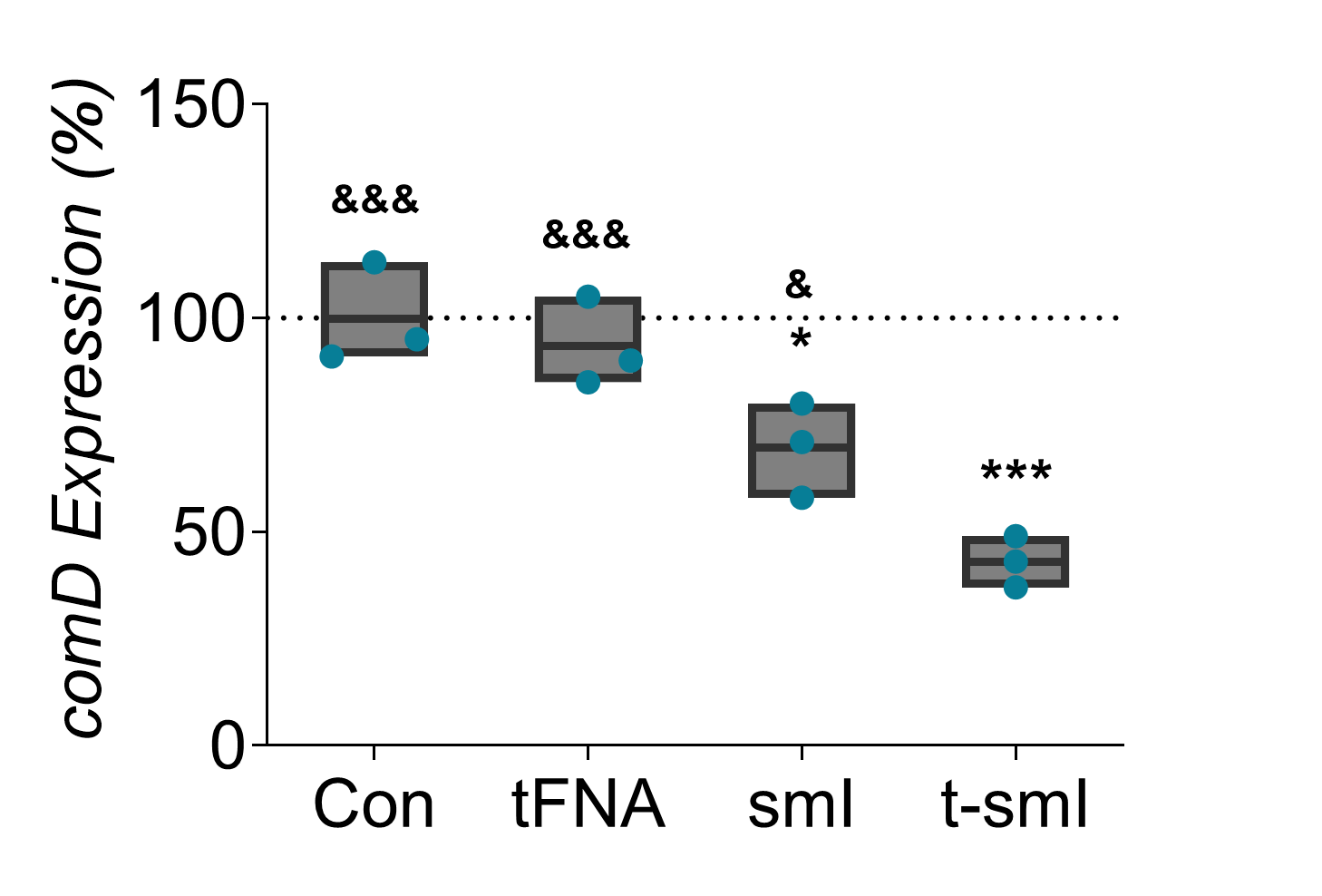


**Fig. S5.** Gene expression of the dual-component signal transduction system. Compared with the Con group, * *p* < 0.05, ** *p* < 0.01, *** *p* < 0.001; compared with the t-smI group, ^&^ *p* < 0.05, ^&&^ *p* < 0.01, ^&&&^ *p* < 0.001; NS, no statistical difference.


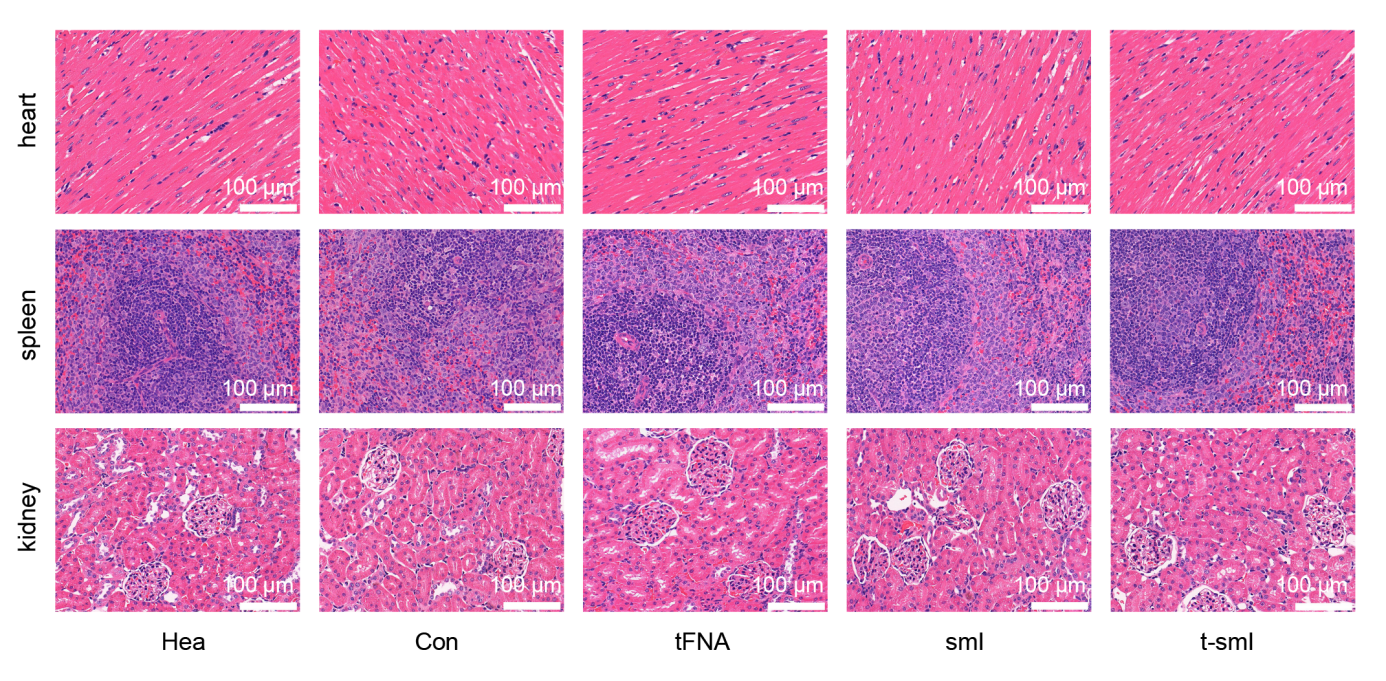


**Fig. S6.** HE histological observation of the heart, spleen, and kidney. Scale bar = 100 nm.
